# Splenectomy revisited in Wiskott–Aldrich syndrome in the era of HSCT and gene therapy: a bridge, a rescue, or simply obsolete?

**DOI:** 10.3389/fimmu.2026.1851771

**Published:** 2026-07-22

**Authors:** Youssuf Khanafer, Ahmad Al-Naemi, Layan Khayyat, Farhan Cyprian

**Affiliations:** 1 College of Medicine, Qatar University (QU) Health, Qatar University, Doha, Qatar; 2 Department of Basic Medical Sciences, College of Medicine, QU Health, Qatar University, Doha, Qatar

**Keywords:** gene therapy, hematopoietic stem cell transplantation, hypersplenism, infection, platelet count, splenectomy, Wiskott-Aldrich syndrome, X-linked thrombocytopenia

## Abstract

Wiskott–Aldrich syndrome (WAS) is a rare X-linked primary immunodeficiency caused by mutations in the WAS gene, classically presenting with microthrombocytopenia, eczema, and recurrent infections, with an incidence of approximately 1 per 100, 000 live births. At the mechanistic level, deficiency of the WAS protein (WASp) disrupts actin cytoskeleton remodeling across hematopoietic cells, impairing immune synapse formation, T-cell receptor signaling, natural killer cell cytotoxicity, B-cell activation, and dendritic cell migration. Concurrently, these cytoskeletal defects compromise megakaryocyte proplatelet formation, producing structurally fragile platelets that are rapidly cleared by the spleen and further targeted by immune-mediated destruction, culminating in thrombocytopenia. This pathophysiological basis explains the historical use of splenectomy to control life-threatening bleeding in refractory cases, as removal of the spleen reduces platelet sequestration and destruction, leading to reliable increases in platelet counts, often exceeding 150 × 10^9^/L, and improved bleeding outcomes. However, the durability of this response is phenotype-dependent, with sustained benefit more common in X-linked thrombocytopenia and relapse occurring in up to 56% of classical WAS cases within the first year. Despite these hematologic benefits, splenectomy does not correct the underlying immune defect and is limited by a substantial risk of overwhelming infection, necessitating lifelong antibiotic prophylaxis, vaccination, and often immunoglobulin replacement. Consequently, with the advent of hematopoietic stem cell transplantation (HSCT) and gene therapy as curative approaches, the role of splenectomy has markedly declined and is now largely restricted to specific scenarios, including as a bridge to HSCT in severe thrombocytopenia, a rescue option when definitive therapies are inaccessible, and a post-HSCT intervention for persistent thrombocytopenia due to mixed donor chimerism. Nonetheless, global disparities in access to HSCT and gene therapy (driven by donor availability, financial constraints, and health system limitations), preserve a limited but important role for splenectomy in resource-constrained settings, underscoring the need for standardized, evidence-based guidelines to ensure its use only when the anticipated benefits outweigh the risks.

## 1 Introduction

Wiskott-Aldrich syndrome (WAS) is a rare X-linked primary immunodeficiency caused by mutations in the WAS gene located on Xp11.22 (1). The gene encodes the Wiskott-Aldrich syndrome protein (WASp), a key regulator of actin cytoskeleton remodeling in hematopoietic cells, and its deficiency leads to immune dysregulation and hematologic abnormalities (2). First described by Alfred Wiskott in 1937 and later characterized by Robert Aldrich in 1954, WAS is a rare disorder with an estimated incidence of approximately 1 per 100000 live births and predominantly affects males (3). Clinically, it is classically defined by the triad of microthrombocytopenia, eczema, and recurrent infections, along with an increased risk of autoimmunity and malignancy (4).

WAS demonstrates a broad clinical spectrum driven by the degree of WASp expression, with clinical manifestations typically arising early in infancy, presenting at a median age of approximately 3 months and commonly diagnosed within the first year of life (5). Classic WAS, associated with absent protein expression, presents with severe immuno-hematologic involvement and a high risk of autoimmunity and lymphoma (6). In contrast, milder phenotypes such as X-linked thrombocytopenia (XLT) exhibit partial WASp expression and a less severe clinical course, although risks of autoimmunity and malignancy remain (7). Rare variants such as intermittent thrombocytopenia have also been described, characterized by fluctuating platelet counts with preserved low mean platelet volume and residual WASp expression (8). Additional phenotypes include X-linked neutropenia (XLN), which is driven by gain-of-function mutations and presents with severe congenital neutropenia alongside an increased risk of myelodysplasia (2, 9). Genotype phenotype correlations show that missense mutations are generally associated with milder disease, whereas non missense variants are linked to more severe presentations (5). Similar WAS-like syndromes may also arise from defects in WASp regulatory proteins such as WIPF1, where loss of WASP-interacting protein (WIP) leads to secondary WASp degradation and a clinically overlapping phenotype (10).

These clinical distinctions have important implications for prognosis and therapeutic decision-making. Historically, WAS was managed with splenectomy and immunoglobulin replacement therapy to reduce infection risk, and although splenectomy can improve platelet counts, it may further compromise immune function and increase susceptibility to severe infections (11). Over time, a major therapeutic shift occurred with the advent of hematopoietic stem cell transplantation (HSCT), first successfully pioneered in 1968 in patients with primary immunodeficiencies, including WAS, using bone marrow from HLA-matched siblings (12). HSCT has since become the cornerstone of curative therapy, providing both immunologic and hematologic correction (13), with reported 3-year overall survival rates approaching 89% (14). More recently, gene therapy has further expanded curative options and improved long-term outcomes, with gene-modifying approaches evolving from γ-retroviral vectors to safer lentiviral platforms and, more recently, to precise genome-editing technologies such as CRISPR/Cas9 and zinc-finger nucleases (1). Despite these advances, WAS remains associated with substantial long-term morbidity and mortality, as demonstrated by large cohorts showing a decline in overall survival from 82% at 15 years of age to 70% by 30 years (15), and access to definitive therapies continues to be limited globally due to donor availability, financial constraints, and health-system barriers.

In this evolving therapeutic landscape, the role of historical palliative interventions requires critical reassessment. Splenectomy has long been employed as a secondary symptomatic-controlling intervention to mitigate life-threatening bleeding complications arising from refractory thrombocytopenia in WAS (16). Although effective in rescuing peripheral platelet counts, this procedure fundamentally aggravates the state of a patient’s partial immunodeficiency, conferring a lifelong, iatrogenic susceptibility to severe and overwhelming infections from encapsulated organisms, necessitating strict adherence to vaccination and antibiotic prophylaxis (17). This significant iatrogenic risk profile, together with the increasing availability and success of curative therapies, necessitates a modern risk–benefit reassessment that renders the role of splenectomy as a standalone long-term management strategy highly questionable.

Consequently, although its role has diminished, splenectomy continues to be considered in specific contexts, primarily as a bridging intervention to stabilize patients prior to HSCT or gene therapy, or as a last-resort option when definitive curative treatments are inaccessible. Nevertheless, a critical evidence gap persists regarding its contemporary outcomes, particularly its impact on subsequent transplant success, long-term infectious morbidity, and overall survival in the era of advanced therapies. Therefore, this paper provides a comprehensive, evidence-based assessment of splenectomy in WAS, examining its historical efficacy, inherent risks, and its evolving, limited indications within modern curative treatment paradigms, with the central aim of clearly defining its precise role in contemporary clinical practice.

## 2 Pathophysiology

### 2.1 Actin cytoskeleton dysfunction in adaptive and innate immune cells

WASp deficiency disrupts actin cytoskeleton dynamics across multiple hematopoietic lineages, leading to widespread immune dysfunction. At a molecular level, WASp functions as a signal-dependent coincidence detector, integrating small GTPase activation (CDC42/Rac), phosphoinositide signaling (PIP_2_), and tyrosine kinase inputs to spatially restrict Arp2/3-mediated actin nucleation to immune synapses (1). In T lymphocytes, impaired actin polymerization results in defective immunological synapse formation, culminating in impaired T-cell receptor signaling (18). This disruption skews adaptive immunity toward a Th2 cytokine profile and compromises cytotoxic and helper T-cell responses, with CD8^+^ dysfunction arising predominantly from cell-intrinsic WASp-dependent defects and further exacerbated by impaired Th1 support. Additionally, WASp is central to lipid raft assembly following TCR/CD28 engagement, and its absence results in reduced calcium signaling and diminished IL-2 secretion, both of which are critical for T-cell activation (19, 20).

Innate immune dysfunction is similarly evident. In natural killer (NK) cells, WASp-dependent actin remodeling is required for immunological synapse formation and cytotoxic function. Its absence impairs raft clustering, conjugate formation with target cells, and F-actin accumulation at the synapse, leading to defective natural and antibody-dependent cellular cytotoxicity, although partial restoration can be achieved with IL-2 treatment (21). In antigen-presenting cells, dendritic cells exhibit impaired podosome formation, reduced chemotaxis, and defective migration to lymphoid tissues, limiting effective antigen presentation and T-cell priming (22, 23). WASp is also essential for the formation and turnover of actin-based structures in dendritic cells and macrophages that regulate adhesion and matrix sensing, and its deficiency impairs chemotactic responses and homing to lymph nodes (20, 24). Furthermore, WASp is required for assembling the actin-rich phagocytic cup, and its absence results in approximately a two-fold reduction in IgG-mediated phagocytosis, particularly during early immune responses (25, 26). Overall, impaired actin-dependent processes disrupt multiple innate immune functions, collectively weakening early immune defense.

Humoral immunity is also significantly affected. In B cells, WASp deficiency disrupts B-cell receptor clustering and trafficking, impairing calcium signaling and leading to defective activation, clonal expansion, class-switch recombination, differentiation, and antibody production (27). B cells from WAS patients exhibit impaired transmembrane signaling, defective polarization, reduced chemotaxis, and impaired spreading (actin-dependent flattening over antigen-presenting surfaces for synapse formation) (24, 28). These defects contribute to reduced marginal zone B-cell populations and poor antibody responses, particularly against polysaccharide antigens, indicating a pronounced impairment in responses to T-independent antigens. Collectively, WASp deficiency compromises both intrinsic B-cell signaling and spatial organization, resulting in functionally immature humoral immunity.

### 2.2 Mechanisms of autoimmunity

Autoimmune manifestations in WAS arise, in part, from defects in immune regulation. Regulatory T cells (Tregs), which rely on proper immunological synapse formation for suppressive function, exhibit impaired activity in the absence of WASp. This defect diminishes their ability to control autoreactive lymphocytes, thereby permitting persistent immune activation and breakdown of peripheral tolerance (24, 29). In addition to Treg dysfunction, abnormalities in regulatory B cells (Bregs) further contribute to immune dysregulation. Evidence from autologous hematopoietic stem-cell gene therapy studies demonstrates that recovery of interleukin-10–producing Bregs, alongside Tregs, is associated with protection against post-treatment autoimmune flares, highlighting their synergistic role in maintaining immune homeostasis (29). These findings underscore the importance of coordinated regulatory networks in preventing autoimmunity. Despite these insights, the precise pathways driving WAS-associated autoimmunity remain incompletely understood. The interplay between defective cytoskeletal signaling, impaired immune synapse formation, and dysregulated regulatory cell populations suggests a multifactorial process, but further mechanistic studies are required to fully elucidate these pathways (30).

### 2.3 Platelet and megakaryocyte defects and hypersplenism

Thrombocytopenia, a defining feature of WAS, results from both impaired platelet production and enhanced peripheral destruction, with peripheral destruction being the predominant mechanism. WASp deficiency disrupts actin-dependent processes in megakaryocytes, impairing their polarization and directional proplatelet extension into bone marrow sinusoids. Experimental models have demonstrated that actin-regulatory proteins, including WASp, ADAP, PFN1, and ARPC2, are essential for proper platelet biogenesis, and their deficiency leads to ectopic release of platelet-like particles within the marrow (31).

As a result, platelets produced in WAS are small, structurally fragile, and exhibit membrane instability and elevated intracellular calcium levels, rendering them highly susceptible to splenic macrophage-mediated clearance. This peripheral destruction is further amplified by immune-mediated mechanisms, including autoantibody formation and hypersplenism, which collectively accelerate platelet turnover (24). Additionally, impaired NK cell function may increase susceptibility to viral infections, indirectly contributing to disease severity and further perturbing hematologic stability (21).

**Figure 1:**
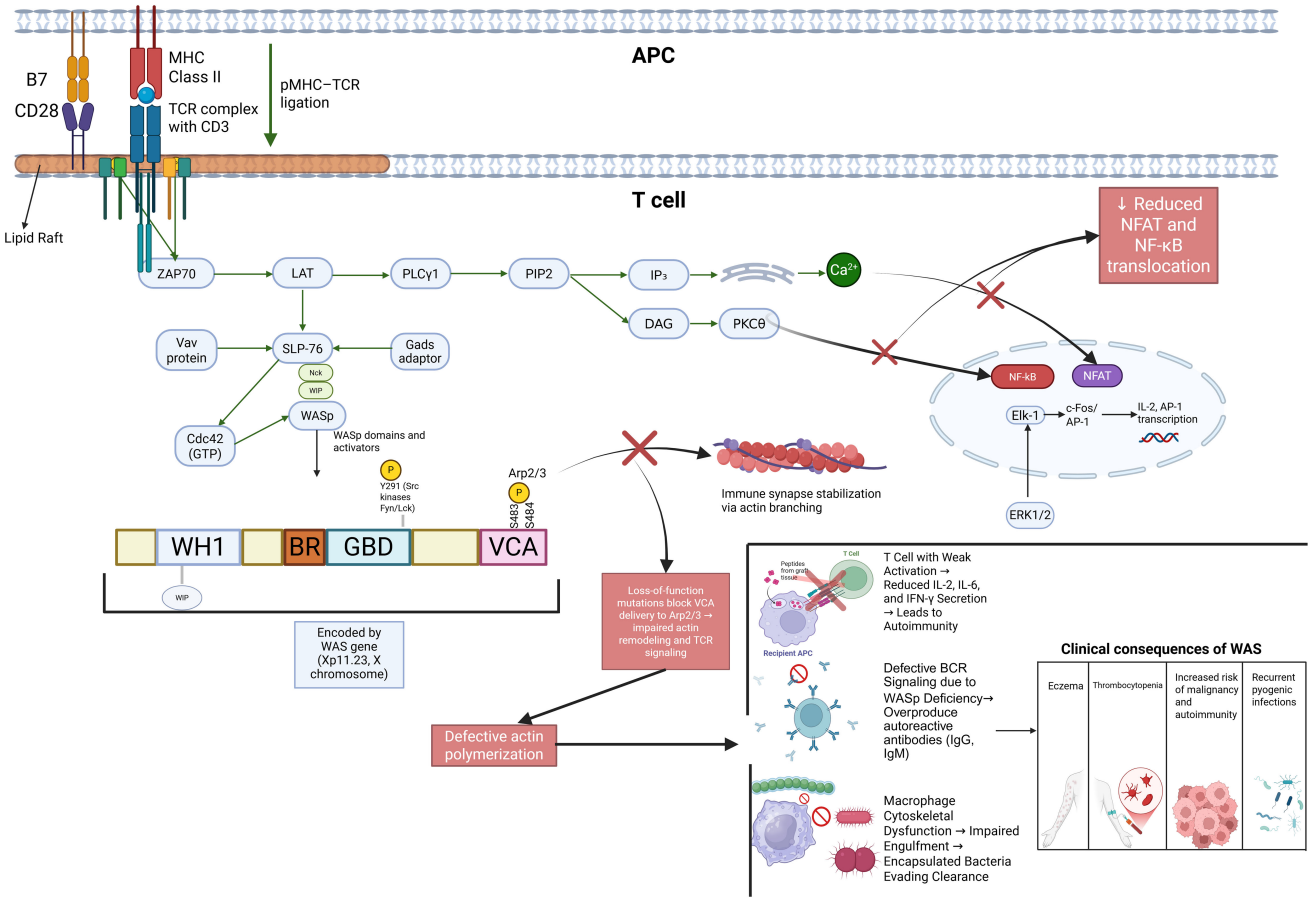
Pathogenesis of Wiskott-Aldrich syndrome. The WAS gene, located at Xp11.23, encodes the WASp. Upon T-cell receptor (TCR) engagement with peptide–MHC complexes, downstream signaling activates LAT and SLP-76, which recruit adaptor proteins (e.g., Nck and WIP) and facilitate Cdc42-GTP binding alongside Src kinase–mediated phosphorylation at Y291 of WASp. This conformational change exposes the VCA domain, allowing WASp to interact with the Arp2/3 complex by delivering actin monomers and promoting nucleation of branched actin filaments, thereby generating a dynamic actin network that stabilizes the immunological synapse and sustains signaling. In WAS, loss-of-function mutations impair VCA–Arp2/3 interactions, leading to defective actin polymerization, unstable synapse formation, and reduced nuclear translocation of NFAT, NF-κB, and Elk-1, resulting in impaired transcription of IL-2 and AP-1–dependent genes. Clinically, this manifests as microthrombocytopenia, recurrent infections, eczema, autoimmunity, and an increased risk of malignancy.

This pathophysiological framework explains why splenectomy is hematologically effective yet immunologically compromising in WAS. Removal of the spleen reduces platelet sequestration and destruction, resulting in rapid and sustained increases in platelet counts. However, this benefit is counterbalanced by impaired immune defense due to loss of marginal zone B cells and splenic macrophages, which are critical for the clearance of encapsulated organisms, thereby compounding the underlying immunodeficiency (24). This trade-off between hematologic improvement and increased infectious risk underpins the role of splenectomy in contemporary clinical decision-making (**Figure 1**).

## 3 Access and availability constraints of curative therapy

**Figure 2:**
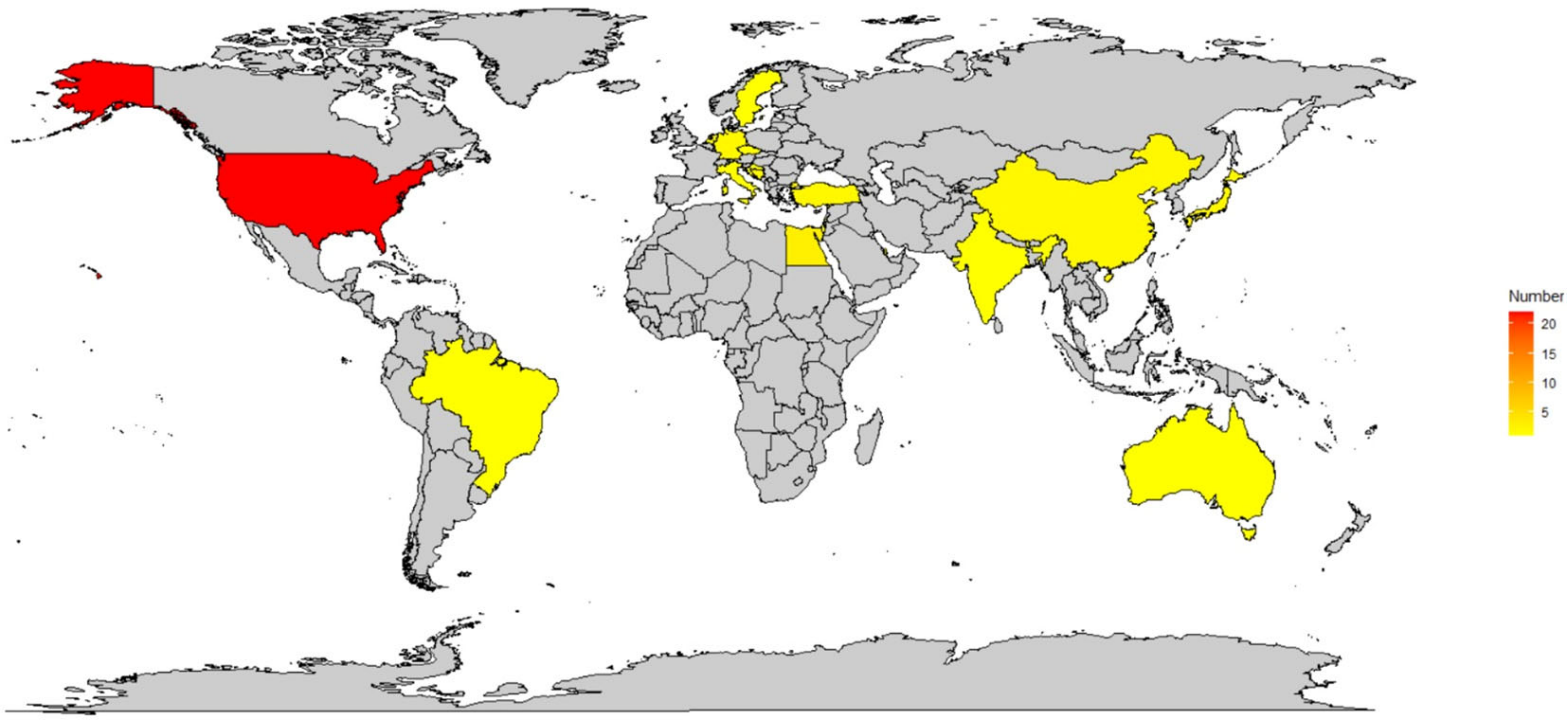
Geographic mapping of cases across the included studies. Geographic distribution of reported cases across the included studies, demonstrating a predominance of cases reported from the United States and Europe, with additional concentrations in East Asia and Australia. Countries are shaded according to the number of reported Wiskott–Aldrich syndrome cases identified in the literature search, with warmer colors indicating a greater number of reported cases. Data were extracted from the studies summarized in **Table 1**. This distribution reflects study reporting patterns rather than true disease epidemiology, as there is no established geographical, ethnic, or regional predominance for Wiskott–Aldrich syndrome.

In low- and middle-income countries, where timely access to HSCT or gene therapy remains limited, splenectomy remains a pragmatic temporizing strategy (**Figure 2**). Despite HSCT being the established curative standard, its access remains uneven in high-income countries and limited or absent in resource-constrained settings. Large registry analysis from Center for International Blood and Marrow Transplant Research (CIBMTR) demonstrate persistent racial disparities in HSCT utilization, with white patients disproportionately represented among allogeneic transplant recipients compared with black and Hispanic patients (32, 33). In part, these inequities are driven by structural donor limitations, whereby minority populations remain severely underrepresented in registries such as the National Marrow Donor Program (NMDP), particularly African American donors (33). This substantially reduces the probability of identifying HLA-matched unrelated donors. Of particular importance, these disparities persist even after donor searches are initiated, indicating that expanding the registry alone does not close the access gap. Beyond donor availability, access to HSCT is further limited by the concentration of transplantation services within a small number of Foundation for the Accreditation of Cellular Therapy (FACT)-accredited centers. The high financial cost of transplantation, along with travel demands and prolonged caregiver commitments, creates additional barriers to timely treatment, particularly for socioeconomically disadvantaged patients, with disparities in access to insurance coverage further limiting it (34, 35).

Beyond the United States, access to HSCT remains markedly limited across many low- and middle-income countries (LMICs), where the complexity and cost of transplantation, together with challenges related to timely diagnosis, donor availability, supportive care, and healthcare infrastructure, continue to restrict access to curative therapies. In Mexico, for example, only approximately 5% of individuals requiring HSCT ultimately receive a transplant (36). Similarly, affordable HLA typing, transplant capacity within public health systems, coverage of transplant-related costs, patient funding, and workforce development remain major determinants of access in LMICs. Notably, the DKMS Access to Transplantation program facilitated 53, 430 HLA typings and 1, 639 transplants, highlighting both the scale of unmet need and the infrastructure required to expand access to curative treatment (37). These disparities are particularly pronounced in Africa, where only 7 of 54 countries currently perform HSCT, with major barriers including inadequate healthcare infrastructure, limited trained personnel, donor scarcity, high costs, cultural misconceptions, and poor documentation (38). Collectively, these findings underscore the substantial global inequities in access to HSCT and the persistent challenges facing resource-constrained health system.

**Figure 3:**
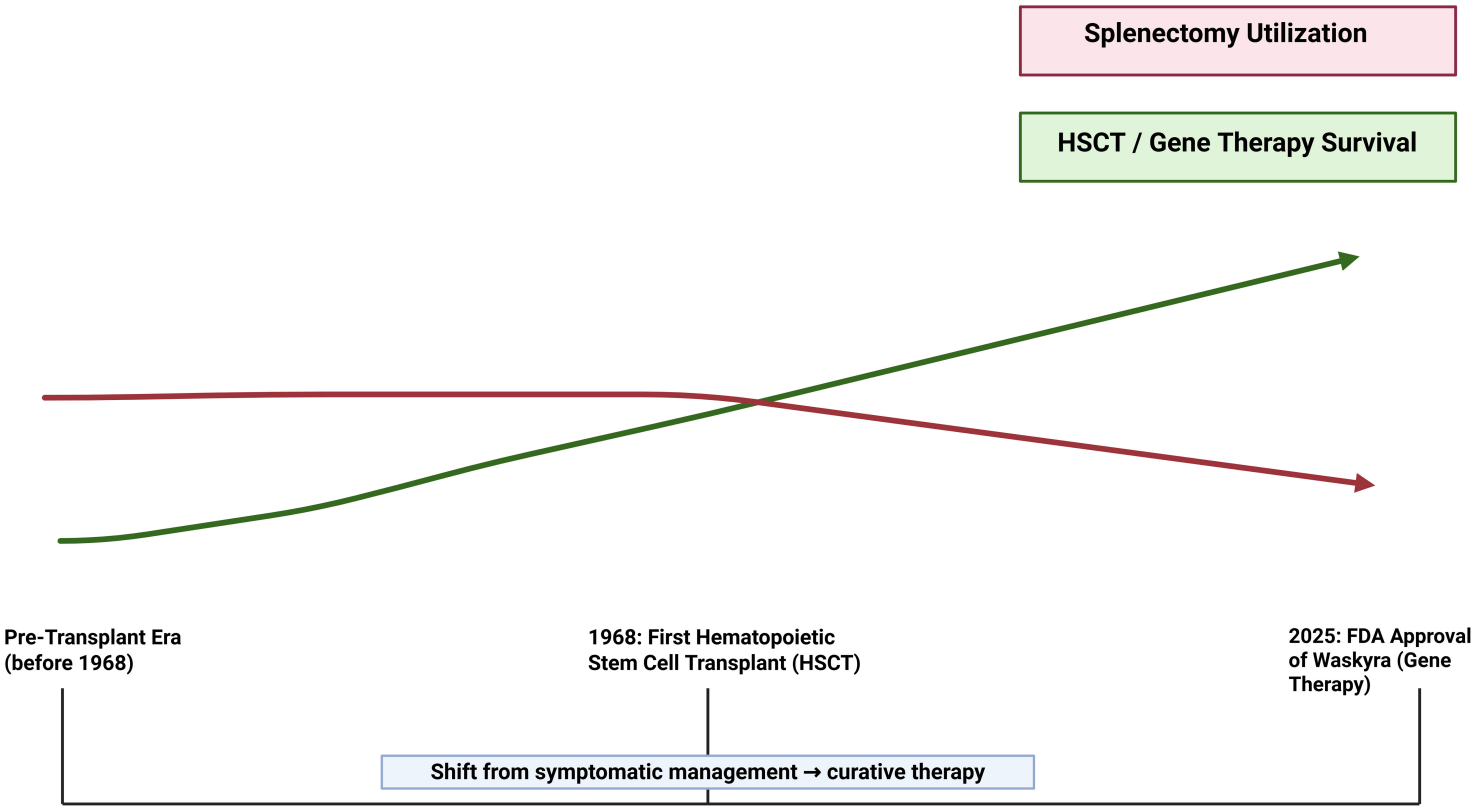
Therapeutic evolution in Wiskott–Aldrich syndrome from splenectomy to curative therapies (Pre 1968–2025). This schematic timeline illustrates the transition from symptomatic management to curative treatment strategies. The red trajectory represents the declining utilization of splenectomy, whereas the green trajectory represents improving survival outcomes following hematopoietic stem cell transplantation (HSCT) and, more recently, gene therapy. Based on historical developments and outcome trends reported in the studies included in this review, the figure highlights the progressive shift from supportive interventions toward curative therapies, culminating in the 2025 FDA approval of Waskyra.

These barriers have been particularly pronounced in the management of WAS, helping contextualize the persistence of non-curative interventions in contemporary practice as well as pushing for standardization of practice (**Figure 3**). In a cross-sectional analysis of WAS patients in the United States, it was demonstrated that inpatient WAS care is highly centralized, with a small fraction of hospitals accounting for a disproportionate share of admissions, with only 10% of hospitals accounting for 42% of all WAS admissions, reflecting referral to transplant-capable centers but also imposing geographic and economic barriers to care (39). Their analysis further identified racial inequities in access to curative transplantation, with African American patients comprising 18.3% of the non-transplant cohort compared with 6.8% of the transplant group. Important to note, those who did not receive early curative therapy exhibited higher long-term healthcare utilization and accumulated complications such as autoimmunity and lymphoma, which were observed exclusively in the non-transplant group (39). This underscores the paramount importance of equitable access to curative therapy. Together with broader evidence linking socioeconomic disadvantage and minority status to delayed referral, reduced transplant uptake, and worse post-transplant outcomes (40, 41), these findings point to persistent inequities in access to care, with White patients remaining overrepresented among recipients of advanced cellular therapies. However, these studies more clearly demonstrate disparities in access and socioeconomic risk than a direct racial difference in post-transplant survival. Access to gene therapy for WAS remains restricted even after approval. The December 2025 FDA approval of Waskyra (etuvetidigene autotemcel), a first-in-class autologous gene therapy for WAS, limits its use to patients without a suitable HLA-matched related donor (42), consistent with the 2025 EBMT indications recommendations that continue to prioritize allogeneic HSCT when such a donor is available (43). Consequently, the use of splenectomy (despite the modern shift away from it), must be critically contextualized as a context-dependent intervention that may be considered when access to definitive curative therapy is delayed, limited, or unattainable.

## 4 Clinical outcomes of splenectomy in Wiskott-Aldrich syndrome

A narrative literature review was conducted using PubMed, Embase, and the Cochrane Library to identify studies evaluating splenectomy in WAS. Given the relative lack of large-scale evidence of the condition, all study designs, from case reports to population-based studies, were considered. Findings were synthesized descriptively, focusing on hematologic outcomes, infection risk, survival, quality of life, and timing relative to HSCT (**Table 1**).

**Table 1:** Clinical outcomes and platelet responses following splenectomy in Wiskott–Aldrich syndrome across eras.

Era	Demographics and procedure details	Platelet response post-op (x 10^9^/L)	Key outcomes	Reference
Pre-2000	47 patients (16 splenectomized); age 15 mo-22 y; indication: thrombocytopenia; prophylaxis: penicillin and sulfisoxazole	262	6 sepsis deaths (mostly without prophylaxis); 1 relapse; 1 brain tumor death; 1 hemorrhage death; 9 received prophylaxis	(Lum et al., 1980) (44)
	19 mo; splenectomy (hemorrhage); prophylaxis: TMP-SMX (daily) + IVIG (monthly from month 8)	206	Relapse at 6.5 mo (controlled with vincristine + steroids); no deaths; no infections	(Knutsen et al., 1981) (45)
	14 WAS (7 splenectomized); indication: platelet/MPV normalization; prophylactic antibiotics	NS*	2 relapses (thrombocytopenia + elevated PA-IgG); no deaths; no infections.	(Corash et al., 1985) (46)
	9 y; splenectomy (~3 y, bleeding), followed by HSCT	NS	No infections; no thrombocytopenia relapse; successful HSCT; later splenosis (abdominal mass)	(Mathurin & Lallemand, 1990) (47)
	Male monozygotic twins; splenectomy at 7 y (one twin); Underlying diagnosis: Attenuated variant of WAS	NS	Twin 1: pneumococcal sepsis (day 10 post-op, survived); died 3 y later (fulminant infection)	(Standen et al., 1990) (48)
	5 WAS (2 splenectomized at 13 mo, 2.5 y); indication: bleeding/hypersplenism; pneumococcal vaccine pre-op; antibiotics	Case 1: NS Case 2: 350	Both survived 1 year; no post-op bleeding or sepsis; Case 2: 3–18 to 350 ×10^9^/L; Case 1: post-op count not stated, but no transfusions needed	(Harfi et al., 1992) (49)
	62 WAS (39 splenectomized); mean age 5.2 y; indication: thrombocytopenia; prophylaxis: TMP-SMX or amoxicillin (post-1978)	262.8	12 developed sepsis (5 fatal); 2 deaths (post-splenectomy bleeding/ITP); 22/39 alive (median 25-y follow-up)	(Mullen et al., 1993) (50)
	8 mo; IVIG ineffective, remission with high-dose IVIG	100 → >200 (after high-dose IVIG)	No infections; platelet increase with high-dose IVIG	(De Martino et al., 1994) (51)
	21 WAS (15 splenectomized); median age 48 mo; no HSCT; received IVIG, antibiotics, and vaccines	232	3 deaths (2 cerebral B-cell lymphoma; 1 progressive multifocal leukoencephalopathy); 4 severe infections; deaths not attributed to splenectomy	(Litzman et al., 1996) (52)
2000-2015	21 y; splenectomy (pre-cardiac surgery); prophylaxis: co-trimoxazole, vaccines, azathioprine	327	No bleeding or infections; alive at 2-y follow-up	(Johnston et al., 2001) (53)
	≥10 WAS/XLT (5 splenectomized); age 17–38; elective splenectomy; preoperative vaccination	173–271	No bleeding, infection, or death post-surgery; follow-up ≤ 2 y	(Joshua et al., 2001) (53)
	507 WAS; splenectomy (45 centers) for platelets <20, 000/mm³ or bleeding; always followed by prophylaxis	NS	Risk of fatal sepsis acknowledged; survival and infection rates NR	(Conley et al., 2003) (54)
	11 Chinese WAS (2 splenectomized); indication: thrombocytopenia; all received monthly IVIG + antibiotics	130-180	1 of 2 splenectomized patients died (graft failure); the other survived with no bleeding recurrence	(Lee et al., 2008) (55)
	96 WAS undergoing HSCT; 28 (29%) had splenectomy before transplant; penicillin used for prophylaxis	NS	2 of 28 splenectomized patients died from post-HSCT infections; 6 developed severe infections; splenectomy linked to increased infection risk after HSCT	(Ozsahin et al., 2008) (56)
	173 XLT; 41 (23.7%) splenectomized (thrombocytopenia/bleeding); median age 7 y (10 mo–43 y)	>100 (7/13 patients)	2 deaths post-splenectomy (sepsis, salmonellosis); 3 severe infections (1 fatal) occurred without antibiotic prophylaxis; 2 severe bleeding episodes post-op	(Albert et al., 2010) (7)
	24 y; splenectomy at 9 y (refractory thrombocytopenia and bleeding); lifelong phenoxymethylpenicillin; vaccinated (pneumococcus, Hib, meningococcus C)	235	No recurrence of thrombocytopenia or severe infections; over 15-y of follow-up	(Syrigos et al., 2011) (57)
	29 patients; pre-HCT splenectomy; mean age NS (range within cohort: 2–240 mo); splenectomy performed for thrombocytopenia.	Post-splenectomy: 95.5 Post-splenectomy + HSCT: 318.3	3/28 splenectomized developed fatal sepsis (meningococcal/pneumococcal) post-HCT; Post-HCT splenectomy performed in 5 patients with persistent thrombocytopenia, achieving mean platelet count of 172.8	(Moratto et al., 2011) (58)
	8 y; WAS + IgA nephropathy; splenectomy (refractory thrombocytopenia); lifelong TMP-SMX	NS	Improved renal function and remission of proteinuria; reduced thrombocytopenia-related complications; no deaths reported	(Liu et al., 2013) (59)
2015-Present	24 XLT (1990–2011); 2 splenectomized pre-HSCT (thrombocytopenia)	NS	Both splenectomized died (post-HSCT sepsis: pneumococcal and pseudomonas); splenectomy increased infection risk (especially with GVHD); better survival in non-splenectomized	(Oshima et al., 2015) (17)
	575 WAS; 78 (14%) splenectomized; mean follow-up 7.4 y; prophylaxis not systematically described	159	Platelet improvement post-splenectomy; 77% reported good/very good QoL; bleeding incidence remained high over long-term follow-up (61% at 30 years)	(Glasmacher et al., 2016) (60)
	25 y male (XLT/WAS); splenectomy post-renal transplant; prophylaxis: acyclovir, TMP-SMX, sulfanilamide; monthly IVIG (5 mo)	150	No severe infections or bleeding; developed cytomegalovirus viremia and cellulitis (resolved with treatment)	(Kai et al., 2016) (61)
	141 pediatric; splenectomy (median age 8.8 y); 2 with WAS, but only the second was described: 6.1 y; ~4 y follow-up; pre-op vaccination given; penicillin prophylaxis initiated later	NS	Patient developed multiple episodes of pneumococcal sepsis (4 sepsis episodes); death at age 10 due to this complication	(Luoto et al., 2016) (62)
	Male XLT; splenectomy at 4 y (thrombocytopenia and bruising); monthly IVIG post-op	150-175	Persistent minor bleeding (e.g. nosebleeds, gum bleeds) post-splenectomy; eosinophil count increased over time	(Kim et al., 2019) (63)
	102 patients (68 classical WAS, 34 XLT); 19 splenectomized (10 XLT, 9 WAS); age 7 mo–14.5 y; all vaccinated pre-op; protective antibodies confirmed in all tested	XLT: >100	56% relapse in classical WAS (often within 1 y); no major infections reported due to adherence to prophylaxis	(Rivers et al., 2019) (64)
	129 WAS (2005–2015); 8 pre-HSCT splenectomy (severe thrombocytopenia); most received myeloablative conditioning	NS	No deaths among splenectomized; overall survival 91% at 5 y; HSCT improved platelet recovery in both groups	(Burroughs et al., 2020) (65)
	197 post-HSCT (2006–2017); 2 splenectomized (persistent thrombocytopenia); both had treosulfan conditioning and mixed chimerism	NS	No deaths among splenectomized; lifelong complications and mixed outcomes noted; splenectomy used for persistent thrombocytopenia in mixed chimerism cases	(Albert et al., 2022) (14)
	2 adult Italian WAS brothers; 1 splenectomized at 5 y → HSCT at 23 y; 1 splenectomized at 8 y → gene therapy at 27 y	Case 1: 49-142 Case 2: 129-154	Temporary platelet stabilization post-splenectomy; no correction of immune dysfunction; both patients improved long-term after HSCT/gene therapy	(Consiglieri et al., 2022) (66)
	17 patients (9 WAS, 8 XLT); age NS; splenectomy (all pre-2000); median time post-diagnosis: 7 mo	186	4 deaths (23.5%): 3 WAS, 1 XLT (due to sepsis and herpetic encephalitis); all splenectomies pre-2000; increased infection risk noted	(Soresina et al., 2025) (67)

*Not specified.

The traditional clinical distinction between classic WAS and XLT has historically guided treatment decisions, with milder phenotypes often managed conservatively. However, recent findings have challenged this binary classification by demonstrating that the boundary between WAS and XLT is far more permeable than previously recognized (68). In their nationwide French cohort, over 38% of patients initially classified as having mild WAS/XLT (severity score ≤3 at age 2 years) later progressed to a score of 4 or 5, developing life-threatening complications such as severe infections, autoimmunity, vasculitis, or malignancies well into adulthood. Crucially, overall survival at 40 years was 73% in mild WAS patients, only modestly better than the 65% observed in severe WAS, and not statistically different (68). These findings underscore a paradigm shift in the understanding of WAS and XLT. Initial clinical severity scores may not reliably predict long-term outcomes, whereas mutation genotype (missense vs. nonsense/splice variants) and residual WASP expression appear to provide greater prognostic value. Therefore, even patients presenting with an XLT-like phenotype require lifelong surveillance and should be considered for definitive therapy (HSCT or gene therapy) before irreversible complications accrue.

Splenectomy has historically been utilized in WAS as a pragmatic strategy to address severe thrombocytopenia and bleeding, particularly in settings where curative options such as HSCT or gene therapy were unavailable or delayed. Across earlier cohorts, splenectomy was consistently associated with substantial improvements in platelet counts and reduction in bleeding events, often transforming transfusion-dependent patients into clinically stable individuals. However, these hematologic benefits were offset by a persistent risk of severe infections, including fatal sepsis, especially in the absence of adequate antibiotic prophylaxis and vaccination. Over time, outcomes improved with the introduction of standardized prophylactic measures and adjunctive therapies such as intravenous immunoglobulin (IVIG), although infection risk remained a defining limitation. As summarized in **Table 1**, the overall evidence reflects a clear trade-off between hematologic efficacy and infectious complications, with more recent cohorts demonstrating better risk mitigation but not complete elimination of these concerns.

### 4.1 Hematologic response and phenotype-specific durability

Splenectomy consistently produces a rapid and substantial rise in platelet counts in WAS, representing its most reliable hematologic benefit. Across decades of literature, this response has been remarkably uniform, with early cohort studies demonstrating increases from severely thrombocytopenic levels (as low as 20 ×10^9^/L) to post-operative means exceeding 250 ×10^9^/L, and most patients achieving counts above 100 ×10^9^/L (44). Contemporary data corroborate these findings, with one cohort reporting a rise to 95.5 ×10^9^/L post-splenectomy and further improvement to 318.3 ×10^9^/L following HSCT, highlighting the additive benefit of definitive immune reconstitution (58). Improvements extend beyond quantity, with normalization of mean platelet volume reported, addressing the characteristic microthrombocytopenia of WAS (46). Subsequent cohort and prospective studies have consistently replicated rapid platelet recovery, often within days, with sustained responses observed across diverse settings, including delayed splenectomy after incomplete post-HSCT correction (52, 58, 67). Larger series and case reports similarly confirm durable platelet stabilization over extended follow-up, with trajectories summarized in **Table 1** (45, 50, 53, 57, 69). Collectively, these findings establish splenectomy as a highly effective intervention for correcting thrombocytopenia in WAS, although HSCT remains the primary modality capable of achieving sustained and definitive hematologic normalization (60, 70). Beyond splenectomy, other pharmacological options documented for successful management of thrombocytopenia in WAS patients include the TPO receptor agonists romiplostim and eltrombopag, which showed overall platelet response rates of 79% and 50%, respectively (71).

Importantly, the durability of this hematologic correction is not guaranteed and varies significantly according to disease phenotype, particularly between classical WAS and XLT. Prospective data from the IPINet cohort demonstrate that splenectomy is more frequently performed in XLT patients (32%, 8/25) compared to classical WAS patients (9.8%, 9/92), reflecting its role as a more definitive option in milder disease (67). This distinction translates into divergent long-term outcomes: in a pivotal study of 19 splenectomized patients, all individuals with XLT maintained stable platelet counts indefinitely, whereas 56% of those with classical WAS relapsed, typically within the first year (64). Thus, while splenectomy may function as a durable solution in XLT, it often represents only a temporary measure in classical WAS, where relapse frequently necessitates subsequent HSCT. This high relapse rate reflects persistent systemic immune-mediated platelet destruction occurring outside the spleen despite its removal (72, 73). Supporting this, other studies report only partial platelet recovery following splenectomy, with complete normalization achieved only after HSCT, underscoring the limitations of splenectomy as a standalone intervention in severe disease (60).

This phenotypic disparity in durability is rooted in the underlying pathophysiology. While splenectomy reduces peripheral platelet destruction, it does not correct the fundamental immune dysregulation driving WAS (74). This limitation is particularly evident in classical WAS, where autoimmune manifestations occur in approximately 26–72% of patients (30), reflecting profound immune dysfunction. Absent or markedly reduced WASp expression disrupts regulatory T-cell homeostasis and skews Th1/Th2 balance, generating a pro-inflammatory cytokine milieu that likely undermines sustained hematologic recovery (75, 76). Consistent with this, patients developing post-HSCT autoimmunity exhibit lower donor chimerism across immune lineages, linking residual host immunity to poorer outcomes (58). In contrast, XLT is typically associated with hypomorphic missense mutations that preserve partial WASp function, allowing more intact cytoskeletal remodeling and immune synapse formation, thereby supporting a more stable immunologic baseline. IPINet data further reinforce this distinction, showing that 88% of XLT patients harbor missense mutations, whereas classical WAS is characterized by more severe mutation types (67). Together, these findings explain why splenectomy provides sustained benefit in XLT but limited long-term efficacy in classical WAS, where unresolved immune dysregulation continues to drive disease progression.

### 4.2 Infection risk in splenectomy

Despite its hematologic benefit, the risk of severe infection remains the principal limitation of splenectomy and a major reason for its declining use. This vulnerability arises from loss of the spleen’s key protective functions, particularly phagocytic clearance of bloodborne pathogens and antibody-mediated defense against encapsulated organisms (77, 78). In patients with underlying immunodeficiency such as WAS, splenectomy therefore confers a persistent susceptibility to overwhelming infection. This risk is not unique to WAS; in the NIH cohort of patients with autoimmune lymphoproliferative syndrome (ALPS), 41% of splenectomized patients experienced sepsis and 6 died from infectious complications, highlighting the potentially devastating consequences of asplenia in immunologically vulnerable populations (79). Given these concerns, the role of splenectomy has progressively diminished over time. In the IPINet study, all splenectomies were performed before 2000, underscoring how this approach has largely been replaced in the modern era by curative therapies such as HSCT and gene therapy (67).

Immunodeficiency in WAS confers a baseline susceptibility to severe bacterial, fungal, and viral infections, particularly from opportunistic pathogens such as cytomegalovirus, herpes simplex virus, Epstein–Barr virus, adenovirus, and Pneumocystis jirovecii, a vulnerability that is further amplified following splenectomy (1). The removal of splenic immune function exacerbates impaired pathogen clearance and antibody-mediated defense, thereby intensifying the risk of overwhelming infection. This heightened vulnerability is consistently reflected in mortality data across eras. In early cohorts predating standardized prophylaxis, fatal post-splenectomy sepsis was frequent, with 6 sepsis-related deaths reported among patients not receiving antibiotic prophylaxis, compared to only 2 non-infectious deaths in those who did (44). Although prophylactic strategies have reduced this risk, they have not eliminated it; infection rates remained substantial, with 33% of patients developing infections without prophylaxis versus 16% with prophylaxis, both groups including fatal outcomes (7). Contemporary data further confirm the persistence of this risk despite modern care, with 4 of 17 splenectomized patients (23.5%) dying in a prospective cohort, including a case of fatal sepsis and herpetic encephalitis (67), and 3 of 28 patients (10.7%) in a retrospective HSCT cohort succumbing to fulminant meningococcal or pneumococcal sepsis following splenectomy (58). Importantly, this infectious vulnerability is not mitigated by milder phenotypes; a fatal case of pneumococcal sepsis has been reported in a patient with XLT after splenectomy, underscoring that phenotypic severity does not confer protection against post-splenectomy infectious risk.

Adherence to prophylactic regimens is the principal determinant of outcomes following splenectomy and represents the mandatory standard of care in WAS. The effectiveness of splenectomy in mitigating infectious risk is therefore highly contingent on strict, lifelong compliance with combined strategies including antibiotic prophylaxis, vaccination, and immunoglobulin support. Long-term follow-up studies demonstrate that patients who remain fully adherent can maintain sustained protection, with some cohorts reporting infection-free survival extending up to 15 years or more (57, 69). Conversely, even brief lapses in adherence can have catastrophic consequences, and suspected non-compliance with prescribed penicillin has been directly associated with fatal outcomes, including fulminant meningococcal sepsis following HSCT (56). Collectively, these findings establish adherence not as an adjunct, but as the critical factor governing the safety of splenectomy in this population.

**Figure 4:**
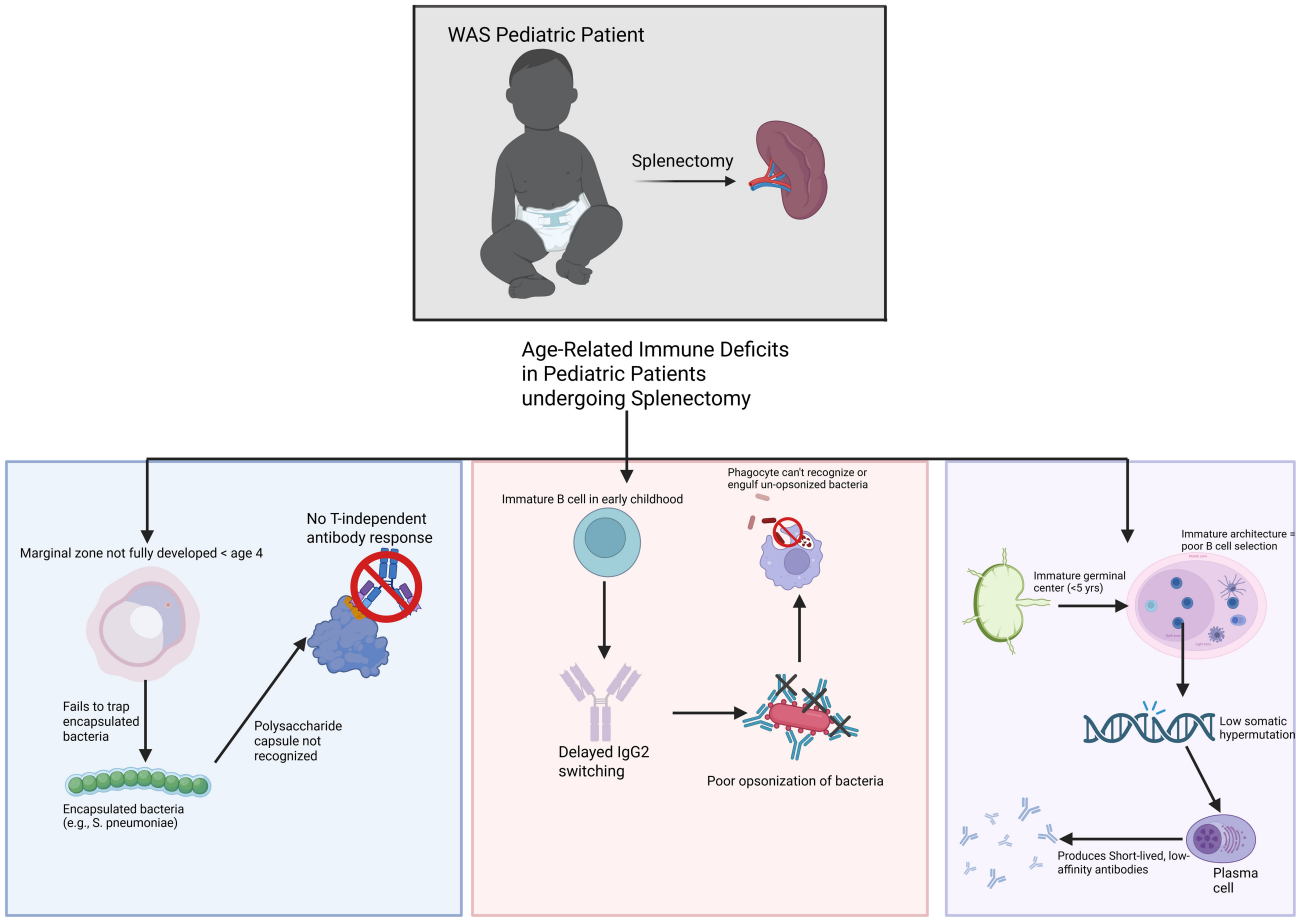
Age-related immune deficits contributing to post-splenectomy infection risk in Wiskott-Aldrich syndrome. In early childhood, incomplete marginal zone development and impaired T-independent antibody responses limit recognition of polysaccharide-encapsulated bacteria. Concurrently, immature B-cell function and delayed IgG2 class switching reduce effective opsonization, impairing phagocytic clearance. Underdeveloped germinal center architecture further results in reduced somatic hypermutation and production of low-affinity, short-lived antibodies. Collectively, these factors contribute to increased susceptibility to severe infections following splenectomy in young patients.

Considering the humoral-immune defects in WAS patients, the immunological rationale for vaccination warrants further elaboration. Given the substantial risk of overwhelming post-splenectomy infection, vaccination against encapsulated organisms remains a cornerstone of prophylactic management. In this context, conjugate pneumococcal vaccines are particularly important, and recent advances have expanded their potential protective benefit. The CDC now recommends the novel 20-valent pneumococcal conjugate vaccine (PCV20) as an option for children under 2 years, providing broader serotype coverage than earlier pneumococcal vaccines (80). The emphasis on conjugate vaccine platforms is particularly relevant in WAS, where vaccine responsiveness is influenced by underlying defects in humoral immunity. While responses to pure polysaccharide vaccines (T-independent antigens) are impaired due to marginal zone B cell hypoplasia and dysfunction (81, 82), conjugate vaccines are pivotal. By covalently linking polysaccharides to protein carriers, these vaccines convert the response to T-dependent. Thus, engaging residual CD4+ T-cell help, which may be partially preserved (particularly in XLT), to promote isotype switching, affinity maturation, and memory B cell formation (6, 83). This active immunization is supplemented by the passive antibody protection provided by IVIG. Prophylaxis becomes particularly more complex with the pediatric population due to developmental immune immaturity, characterized by reduced B-cell reserves and limited IgG2 production (84, 85). Additionally, memory responses from vaccines may be weaker in pediatric patients, necessitating frequent boosting and strict adherence to multi-dose schedules to maintain protective titers. Due to these limitations, conjugate vaccines are especially valuable in children because they convert polysaccharide antigens into T-dependent immune responses, engaging residual CD4+ T-cell help and partially compensating for age- and disease-related defects in humoral immunity (86, 87) (**Figure 4**). Overall, while conjugate vaccines improve antibody responses by engaging residual T-cell help, this protection is often incomplete and short-lived. In clinical practice, this means vaccination alone is insufficient, requiring strict adherence to boosters, immunoglobulin support, and long-term prophylactic management to reduce infection risk in splenectomized WAS patients.

In summary, while prophylactic regimens comprising antibiotics, vaccines, and IVIG can substantially attenuate the infection risk, they do not abolish it. The persistent hazard of overwhelming post-splenectomy infection, especially with encapsulated organisms, necessitates that the decision to proceed must involve a careful risk-benefit analysis, weighing the imperative for hematologic control against the introduction of a permanent and potentially lethal immunologic vulnerability.

### 4.3 Timing relative to definitive stem cell therapy

**Figure 5:**
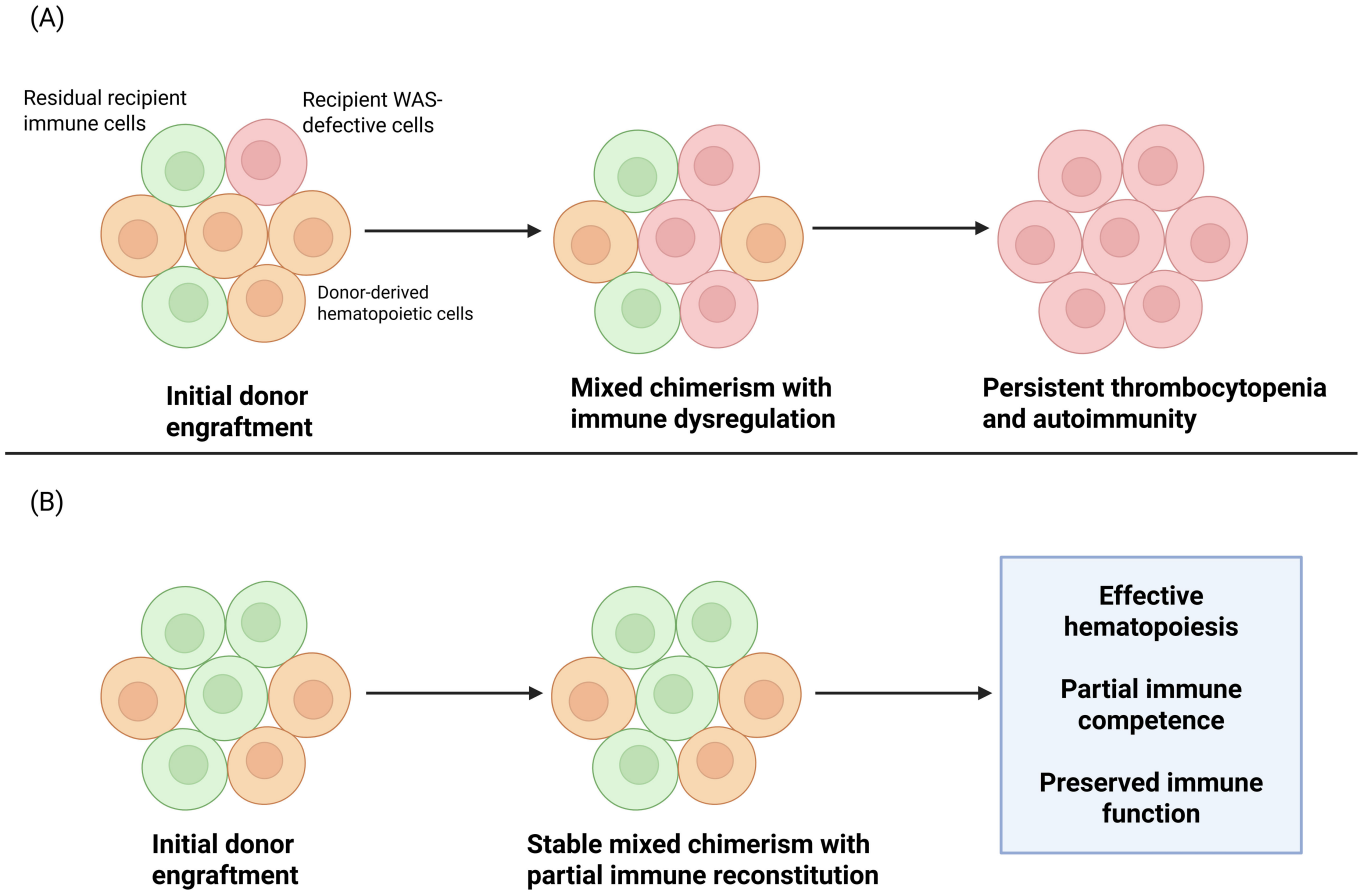
Clinical consequences of mixed donor chimerism following HSCT in Wiskott–Aldrich syndrome. **(A)** Incomplete donor engraftment with persistence of recipient WAS-defective immune cells results in mixed chimerism associated with immune dysregulation, autoimmunity, and ongoing peripheral platelet destruction, leading to persistent thrombocytopenia. **(B)** Stable mixed chimerism with sufficient donor-derived hematopoietic and immune cell contribution supports effective hematopoiesis and partial but clinically adequate immune competence, allowing control of thrombocytopenia despite incomplete donor dominance.

Peri-transplant splenectomy timing is a key determinant of morbidity and mortality in WAS. Pre-HSCT splenectomy is consistently associated with increased infectious risk, as patients are rendered asplenic during the period of peak immunosuppression from conditioning. This exposes them to dual immune insults, combining loss of splenic function with impaired adaptive immunity. In a cohort of 96 WAS patients undergoing HSCT, 2 of 28 (7.1%) pre-splenectomized patients died from post-transplant infections and 6 of 28 (21.4%) developed severe infections, rates higher than in non-splenectomized recipients (56). Similar findings have been reported elsewhere, including deaths from pneumococcal and Pseudomonas sepsis and consistently elevated severe infection rates in this group (17, 56). In contrast, post-HSCT splenectomy, most commonly performed for persistent thrombocytopenia in the setting of mixed donor chimerism, may be associated with improved platelet counts and improved outcomes in selected patients. However, the available evidence is limited by small sample sizes, retrospective study designs, and historical cohorts, precluding strong conclusions regarding its effectiveness. Mixed chimerism, defined by the coexistence of donor and recipient hematopoietic cells, is associated with immune dysregulation and increased autoimmunity (88, 89) (**Figure 5**), while low donor myeloid chimerism (<50%) is a key driver of persistent thrombocytopenia due to impaired platelet production and ongoing splenic sequestration (58, 90).

In this context, post-HSCT splenectomy directly targets peripheral platelet destruction while benefiting from partial donor-derived immune reconstitution, including restoration of immune surveillance and elements of marginal zone function, thereby reducing infectious risk compared with pre-HSCT intervention (91, 92). Notably, this protective effect may persist even in the setting of mixed chimerism because WASp-expressing T cells possess a strong selective advantage, resulting in preferential expansion of donor-derived functional lymphocytes and disproportionate restoration of adaptive immune function relative to overall donor chimerism levels (93). Clinical data support this approach, demonstrating normalization of platelet counts without increased mortality in patients with persistent thrombocytopenia and mixed chimerism (14, 58). However, the optimal donor chimerism threshold for safely performing splenectomy remains undefined. Importantly, timing must also be interpreted in light of disease phenotype. In XLT, splenectomy may serve as a definitive therapy rather than a bridge, particularly when HSCT is not pursued due to a milder immune phenotype. This is reflected in IPINet data, where splenectomy was performed in nearly one-third of XLT patients (67). In carefully selected cases, with strict lifelong prophylaxis, splenectomy alone can achieve sustained platelet normalization and favorable long-term outcomes without transplantation (64), underscoring the importance of phenotypic stratification in guiding both timing and overall treatment strategy.

### 4.4 Long-term outcomes: survival and quality of life

Long-term outcomes following splenectomy in WAS are strongly influenced by treatment era and disease phenotype. In the pre-HSCT era, splenectomy functioned as a life-extending intervention for severe thrombocytopenia, with historical cohorts demonstrating a marked survival advantage, including a median survival of 25 years in splenectomized patients compared with approximately 4 years in those managed without splenectomy (50). However, this apparent benefit must be interpreted in the context of limited curative options at the time. In the modern era, survival is primarily determined by successful HSCT, with approximately 91% of patients alive at 5 years following transplantation (65). Consequently, the role of splenectomy has shifted from a survival-defining intervention to a supportive or adjunctive strategy within a broader curative framework.

Within this contemporary context, the impact of splenectomy on long-term survival, particularly when performed prior to HSCT, remains uncertain. Available evidence suggests that, with stringent infectious prophylaxis, prior splenectomy does not necessarily compromise post-transplant outcomes. In a retrospective cohort of 129 WAS patients undergoing HSCT, all 8 patients who had undergone splenectomy prior to transplantation were alive at last follow-up (median 4.5 years), indicating no evident survival penalty in this subgroup (65). These findings align with broader HSCT outcomes, including a large collaborative study reporting an overall survival of 84.0% that included splenectomized patients (58). However, this must be balanced against persistent long-term risks of asplenia, particularly overwhelming post-splenectomy infection, which remains closely linked to underlying immunodeficiency and contributes to mortality, as illustrated by fatal pneumococcal sepsis despite prophylaxis in pediatric cohorts (62).

Phenotype further refines long-term survival expectations. Patients with XLT consistently demonstrate more favorable outcomes compared with classical WAS. In a large international cohort of 575 WAS/XLT patients, splenectomy was performed in 78 individuals (14%) and was associated with substantial platelet improvement and favorable physician-reported quality of life, although splenectomy-specific survival was not reported (60). Importantly, survival analyses indicate that splenectomy does not significantly worsen long-term survival in XLT compared with non-splenectomized patients (7), likely reflecting the milder intrinsic immune dysfunction of this phenotype. This distinction is reinforced by prospective IPINet data demonstrating an overall survival of 83% at 20 years in XLT populations, including both splenectomized and non-splenectomized individuals (67). In contrast, in classical WAS, durable survival remains dependent on successful immune reconstitution through HSCT, with splenectomy serving primarily as a temporizing intervention rather than a determinant of long-term survival.

**Figure 6:**
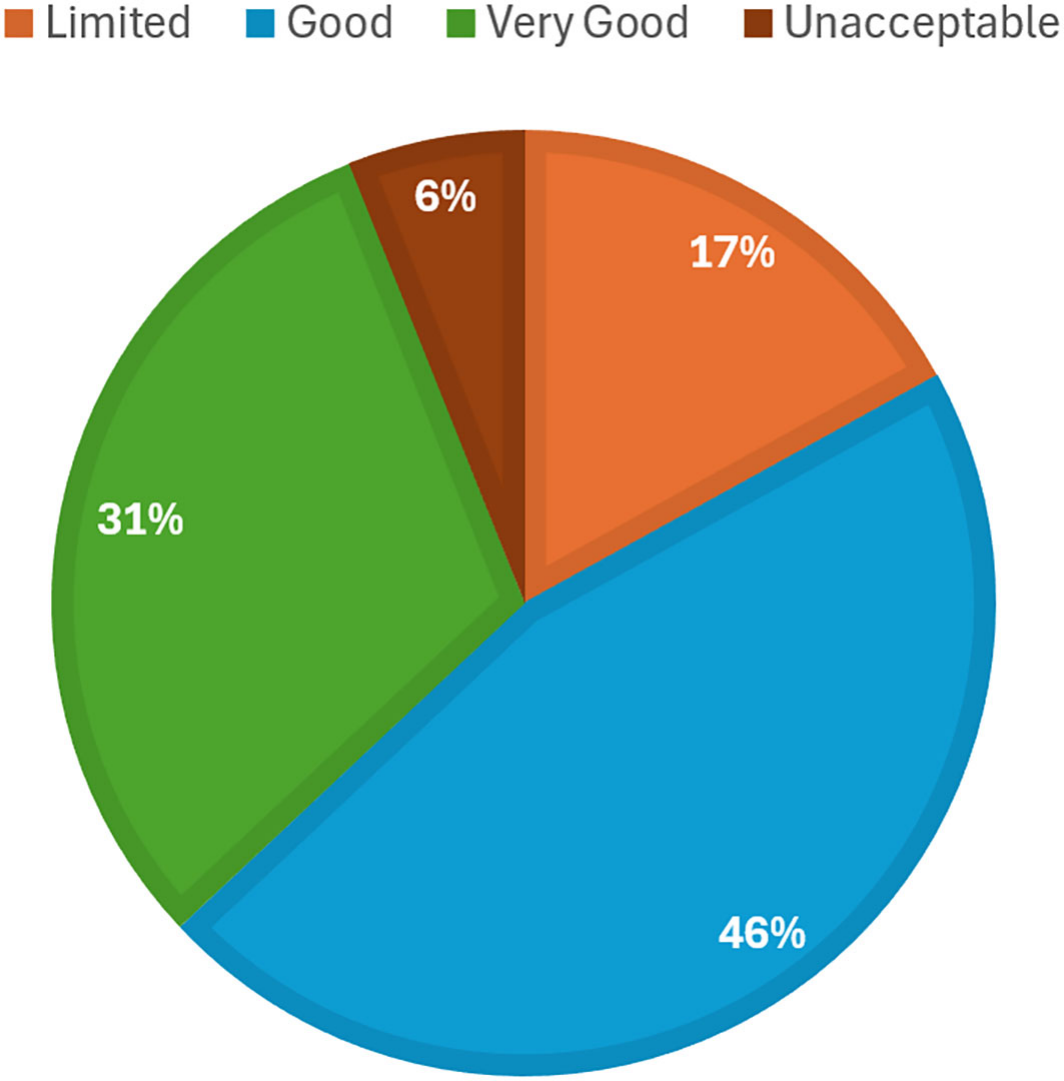
Physician-perceived distribution of quality of life (QoL) outcomes among splenectomized Wiskott–Aldrich syndrome patients (Glasmacher et al., 2016) (60). The figure illustrates that most patients were perceived by their physician to have good or very good quality of life following splenectomy.

Beyond survival, splenectomy has a meaningful impact on quality of life through sustained control of bleeding symptoms. Reductions in epistaxis, gingival bleeding, and bruising alleviate major functional limitations, particularly in pediatric patients, enabling resumption of normal physical activity and long-term stability over follow-up periods exceeding 15 years (50, 57, 69). This benefit is most pronounced in XLT, where patients often achieve unrestricted activity and substantial quality-of-life improvement (64, 94). Consistent with this, physician-reported quality of life was rated as “good” or “very good” in 77% of splenectomized patients (31% “very good”, 46% “good”), with fewer patients reporting limited (17%) or unacceptable (6%) outcomes (60) (**Figure 6**). However, these improvements are largely hematologic and do not extend to the underlying immune dysfunction. Eczema often persists due to ongoing T-cell dysregulation (2, 95), and patients remain at risk for autoimmunity and malignancy, including autoimmune hemolytic anemia, vasculitis, B-cell lymphoma, and Kaposi sarcoma (52, 67). These outcomes reflect persistent defects in immune regulation, including impaired regulatory T-cell function and abnormal immune synapse formation (18, 96), emphasizing that splenectomy improves bleeding-related quality of life but does not modify the broader immunopathophysiology of WAS.

### 4.5 Hypersplenism and the evolution of procedural approaches

Hypersplenism represents an underrecognized but clinically significant contributor to cytopenias in WAS, driven by excessive splenic sequestration and destruction of blood cells. This process can exacerbate thrombocytopenia, anemia, and leukopenia independently of bone marrow function, thereby compounding the hematologic burden of the disease (97). In this context, splenectomy functions as a targeted intervention by eliminating peripheral destruction and restoring circulating cell counts. A representative case illustrates this mechanism in a 2.5-year-old WAS patient with severe hypersplenism and pancytopenia despite preserved megakaryocytes on bone marrow biopsy, whose clinical course was marked by recurrent mucocutaneous and rectal bleeding alongside repeated infections (49). In the absence of a suitable HLA-matched donor, splenectomy was performed following pneumococcal vaccination, resulting in sustained platelet stabilization, cessation of transfusion requirements, and overall clinical improvement aside from mild eczema and occasional febrile episodes. This highlights splenectomy as an effective definitive strategy in settings where curative therapies such as HSCT are unavailable.

Contemporary management of hypersplenism has increasingly explored more conservative and staged approaches, with emphasis on spleen-preserving strategies before considering total splenectomy. Pharmacologic agents including hydroxyurea, thalidomide, and ruxolitinib have been increasingly utilized in selected causes of hypersplenism, although these approaches are not specific to WAS (98, 99). Partial splenic embolization (PSE) represents an additional spleen-preserving interventional option. By inducing controlled infarction through selective occlusion of splenic arterial branches, PSE reduces splenic phagocytic activity while preserving residual immune function, thereby mitigating the risks associated with complete splenectomy. This approach has demonstrated efficacy in improving platelet counts in WAS patients as a bridge to transplantation (100). Furthermore, evidence from broader hypersplenism populations, although not specific to WAS, has shown rapid and sustained hematologic improvement, with platelet counts rising within one week, peaking at one month, and remaining elevated at six months, with additional gains observed following repeat procedures (101). While these findings suggest that PSE may represent a promising spleen-preserving strategy for selected patients with WAS, the available evidence remains extremely limited and further studies are needed to establish its safety and efficacy in this population. More recently, PSE has also been proposed as a preoperative optimization strategy prior to splenectomy, reducing splenic volume, improving hematologic indices, and decreasing intraoperative bleeding risk (102). Collectively, these evolving procedural strategies underscore a shift from definitive splenectomy toward individualized, risk-adapted management of hypersplenism, particularly in patients with delayed or inaccessible definitive therapies such as HSCT or gene therapy. However, much of the evidence supporting these approaches is derived from broader hypersplenism populations rather than patients with WAS specifically, and data regarding their long-term safety and efficacy in WAS remain limited.

### 4.6 Clinical controversies, patient selection, and the imperative for guidelines

**Figure 7:**
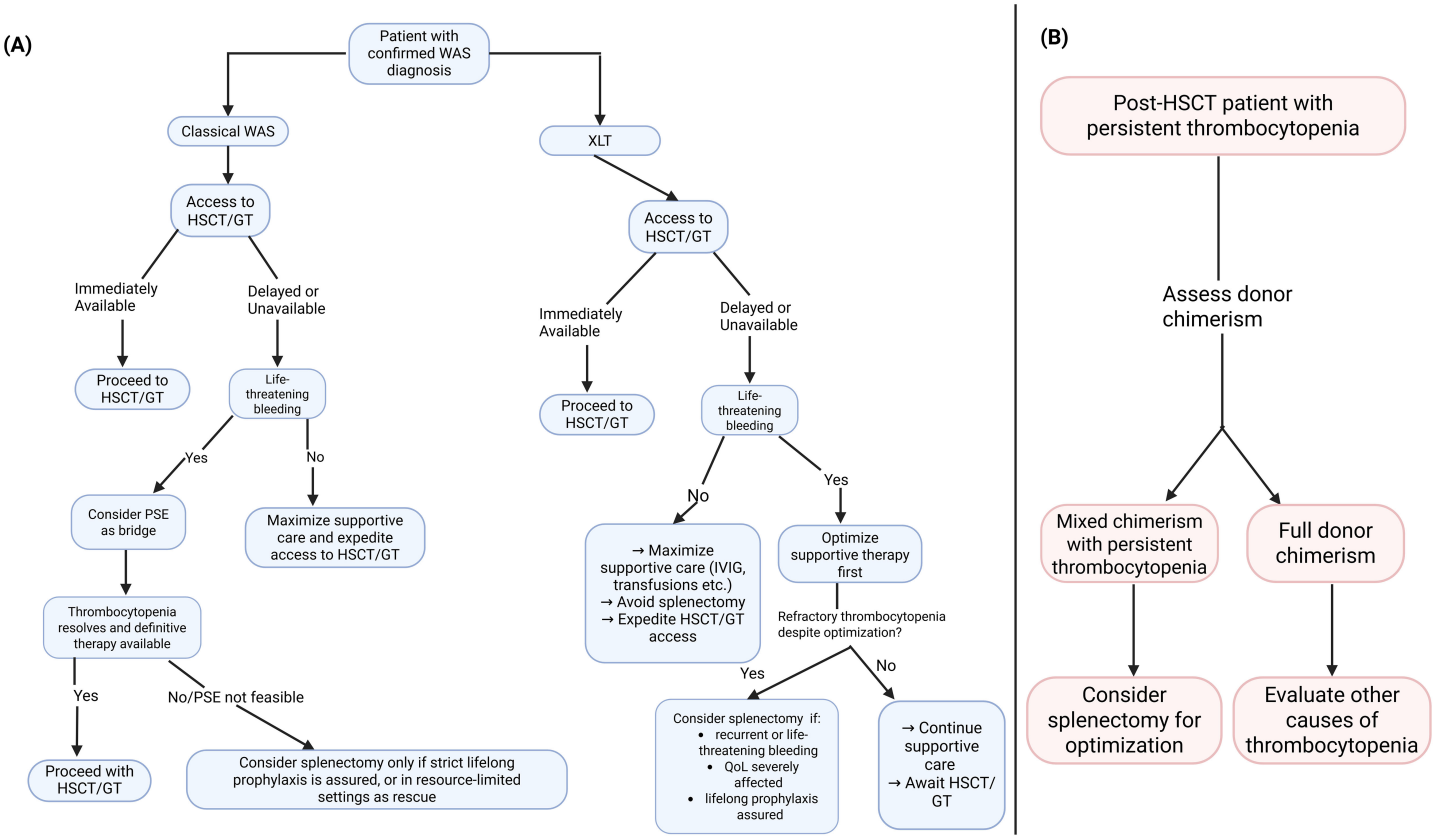
Evidence-based management algorithm for Wiskott–Aldrich syndrome. This proposed algorithm is based on the evidence synthesized in this review and is intended as a conceptual management framework; prospective studies and standardized clinical guidelines remain needed. **(A)** Phenotype-based management pathway prioritizing HSCT or gene therapy as definitive treatment; when delayed or unavailable, care is guided by bleeding severity and response to supportive therapy, with splenectomy reserved for selected refractory cases requiring strict lifelong prophylaxis. **(B)** Post-HSCT management of persistent thrombocytopenia based on donor chimerism, where mixed chimerism may warrant splenectomy for optimization and full donor chimerism prompts evaluation of alternative causes.

The utilization of splenectomy in WAS is now highly selective. A large international survey evaluating practices across 73 centers revealed that only 9.2% routinely performed splenectomy on all WAS patients, 59.2% used it selectively, and 26.3% avoided it altogether (54). The trend away from splenectomy is clearly evidenced in the IPINet prospective study, where the procedure was exclusively performed before the year 2000, and its use has dramatically declined with the advent of safer, curative options like HSCT and gene therapy (67). From a modern clinical perspective, the risks of infectious mortality associated with splenectomy now carry greater weight than its hematologic benefits, particularly in the era of HSCT (39). As early as 2003, high-volume centers already demonstrated a cautious approach to splenectomy, with over 70% using it in 20% or fewer of their cases, reserving it primarily for significant bleeding, absence of planned HSCT, and platelet counts below 20, 000/mm³ (54). Adding on this, in an analysis of 383 pediatric WAS hospitalizations between 2006 and 2012, Agarwal et al. reported no splenectomy procedures in either transplant or non-transplant cohorts (39). This, again, reflects a clear contemporary shift away from splenectomy for WAS-related thrombocytopenia in favor of alternative supportive strategies due to concerns over long-term infectious risk. There is marked inconsistency and a lack of standardized selection criteria for splenectomy in WAS, with factors such as genotype, age, and social context rarely guiding decisions in a uniform manner. Although all centers recognize the risk of fatal sepsis and stress strict post-operative prophylaxis (54), no unified protocol exists, highlighting the need for clear, evidence-based guidance. Based on the available evidence, we propose a structured, evidence-informed clinical framework to support splenectomy decision-making in WAS (**Table 2**; **Figure 7**), integrating key determinants including disease phenotype, bleeding severity, transplant access, chimerism status, and the feasibility of long-term prophylaxis. While this framework provides a structured approach to patient selection, it remains limited by the heterogeneity of existing data and should be interpreted as a hypothesis-generating tool rather than a definitive guideline.

**Table 2:** Potential case-by-case approach for splenectomy in Wiskott–Aldrich Syndrome.

Indication	Preferred phenotype	Access to HSCT/GT	Prophylaxis adherence	Recommended approach
Severe refractory thrombocytopenia with life-threatening or recurrent bleeding	Any	Delayed or unavailable	High (proven ability)	Bridge splenectomy (only after failure of optimized supportive therapy)
Resource-limited setting (no access to HSCT/GT)	XLT	Unavailable	Proven lifelong adherence	Rescue splenectomy (selected cases only)
Post-HSCT persistent thrombocytopenia (mixed chimerism)	Any	Already performed	High	Splenectomy as optimization strategy
Classical WAS (young patient)	Classical WAS	Immediately available	Any	Avoid splenectomy; proceed directly to HSCT or gene therapy

To safely maintain its place in clinical practice, there is an urgent need to develop standardized, prospectively validated guidelines for both patient selection and prophylactic management. A formalized selection tool must integrate multiple variables, including bleeding severity, WAS genotype (classical WAS vs. XLT), chimerism status for post-HSCT decision-making, access to transplant, patient age, and the demonstrated ability to adhere to lifelong prophylaxis. Concurrently, guidelines must standardize optimal prophylactic regimens by defining antibiotic strategies, vaccination schedules, and IVIG thresholds, alongside clear long-term monitoring protocols. Moreover, given the substantial heterogeneity present in the literature, prospective and standardized studies are required to refine and validate the proposed framework and to better define the role of splenectomy as a bridge to definitive therapy. Only through such rigorous standardization can the potential benefit of this historically important, yet increasingly nuanced, intervention be preserved while its substantial risks are meticulously contained.

## 5 Conclusion

In summary, splenectomy occupies a nuanced and increasingly circumscribed role in the modern management of WAS and cannot be considered a definitive therapy. In the post-Waskyra era, it functions strictly as a bridge to curative interventions, a rescue in resource-limited settings, or a targeted fix for persistent post-HSCT thrombocytopenia due to mixed chimerism. While the procedure reliably improves platelet counts and reduces bleeding risk, thereby enhancing quality of life, these benefits are counterbalanced by a lifelong risk of overwhelming infection that is only partially mitigated through rigorous, combined prophylactic strategies. Outcomes remain strongly phenotype-dependent, with XLT patients faring more favorably than those with classical WAS, and are further influenced by timing relative to HSCT, as post-transplant splenectomy (e.g., in setting of mixed chimerism) is generally associated with fewer complications. Accordingly, any decision to proceed must be guided by careful consideration of phenotype, timing, and strict adherence to prophylaxis. The decision to perform splenectomy in a child with WAS can no longer be dictated by local practice patterns, but must rather be aligned with a shared, evidence-based international framework. We urgently call for the establishment of collaborative registries and formal clinical guidelines to standardize patient selection, perioperative management, and long-term follow-up, ensuring that this high-risk intervention is employed only when its life-saving potential clearly outweighs its substantial risks.
